# Supplementary material for: Phenotypic effects of the U-genome variation in nascent synthetic hexaploids derived from interspecific crosses between durum wheat and its diploid relative Aegilops umbellulata
Source: PLoS One. 2020 Apr 2;15(4):e0231129. doi: 10.1371/journal.pone.0231129 (PMC7117738; doi:10.1371/journal.pone.0231129)
Supplement: S1 Table — (DOC) [file pone.0231129.s001.doc]

**S1 Table.** Primers used in this study


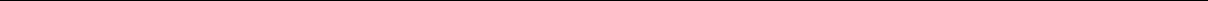


| Marker | Chromosome | Restriction | Annealing |
| --- | --- | --- | --- |
| name |  | Primer sequences (5’-to-3’) | temperature |
|  |  | enzyme |  |
| U925 | 1U | TGTCTGCAGCTCTCGTGGGA | 58°C |
|  |  | – |  |
|  |  | GCTGGACGGGAGACACGTTG |  |
| U929 | 2U | TACCCAGTCCACCCACGGTC | 58°C |
|  |  | *Sfa*NI |  |
|  |  | ACACGCAGCAAGAACTCCCC |  |
| U937 | 3U | ACCAAAGAGGATGACATGTTGCG | 58°C |
|  |  | *Xsp*I |  |
|  |  | ACGGTCACTGAGCACATGCA |  |
| U941 | 4U | GCACAGTGGGTCCTGCCATC | 58°C |
|  |  | *Hae*II |  |
|  |  | ATCAAGGCGGCTTCACGACG |  |
| U973 | 5U | GCCAGTGTCAGAGCGAAAACC | 60 °C |
|  |  | *Tsp*45I |  |
|  |  | ATCCCCACAGGCAGAGTGCT |  |
| U955 | 6U | CCCATCTCGCTTTTGCTGAC | 58°C |
|  |  | *Spe*I |  |
|  |  | TCCGACGGCTTTCAGCACAC |  |
| U961 | 7U | CCGACGGCGTTCCTGTTCTG | 58°C |
|  |  | *Spe*I |  |
|  |  | AGTGCAGGGCAACAGTACCC |  |
