# Supplementary material for: Phenotypic effects of the U-genome variation in nascent synthetic hexaploids derived from interspecific crosses between durum wheat and its diploid relative Aegilops umbellulata
Source: PLoS One. 2020 Apr 2;15(4):e0231129. doi: 10.1371/journal.pone.0231129 (PMC7117738; doi:10.1371/journal.pone.0231129)
Supplement: S2 Table — (DOC) [file pone.0231129.s002.doc]

**S2 Table.** Variations in morphological traits in Ldn, synthetics, and corresponding *Ae. umbellulata* parental accessions.


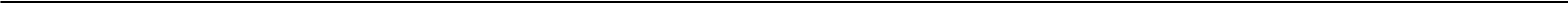


|  |  | ABU hexaploids |  |  |  |  | Parental Aegilops umbellulata accessions | | | |  |  | ABD hexaploids |  |  |  |  | Ldn |  |  |  |  |
| --- | --- | --- | --- | --- | --- | --- | --- | --- | --- | --- | --- | --- | --- | --- | --- | --- | --- | --- | --- | --- | --- | --- |
| Traits |  | Mean | SD | Max | Min | Coefficient of | Mean | SD | Max | Min | Coefficient of Correlation | | Mean | SD | Max | Min | Coefficient of | Mean | SD | Max | Min | Coefficient of |
|  |  |  |  |  |  | Variation |  |  |  |  | Variation | value (r) |  |  |  |  | Variation |  |  |  |  | Variation |
|  |  |  |  |  |  |  |  |  |  |  |  |  |  |  |  |  |  |  |  |  |  |  |
| HD | Heading time (days) | 150.00 | 4.17 | 159.00 | 139.00 | 0.028 | 153.00 | 8.89 | 177.00 | 136.00 | 0.058 | 0.650*** | 149.00 | 4.84 | 156.00 | 142.00 | 0.032 | 153.00 | 3.84 | 157.00 | 149.00 | 0.025 |
| FD | Flowering time (days) | 157.00 | 3.39 | 164.00 | 149.00 | 0.022 | 159.00 | 7.31 | 181.00 | 146.00 | 0.046 | 0.594** | 156.00 | 4.38 | 166.00 | 150.00 | 0.028 | 159.00 | 4.86 | 163.00 | 150.00 | 0.031 |
| FLL | Flag leaf length (cm) | 17.38 | 3.32 | 27.80 | 11.80 | 0.191 | 5.03 | 1.20 | 8.40 | 3.10 | 0.239 | 0.009 | 23.58 | 3.07 | 31.90 | 19.00 | 0.130 | 22.93 | 4.58 | 29.10 | 17.10 | 0.200 |
| FLW | Flag leaf width (cm) | 1.18 | 0.14 | 1.57 | 0.87 | 0.116 | 4.56 | 0.62 | 5.90 | 3.00 | 0.135 | 0.217 | 1.41 | 0.19 | 1.63 | 0.90 | 0.133 | 1.33 | 0.14 | 1.57 | 1.13 | 0.104 |
| LL2 | Leaf length (cm) | 23.55 | 3.64 | 33.70 | 17.10 | 0.155 | 6.93 | 1.53 | 12.00 | 3.70 | 0.221 | –0.184 | 32.72 | 4.15 | 43.00 | 25.10 | 0.127 | 30.55 | 5.41 | 37.20 | 23.90 | 0.177 |
| LW2 | Leaf width (cm) | 1.07 | 0.12 | 1.40 | 0.73 | 0.110 | 4.41 | 0.50 | 5.70 | 3.20 | 0.112 | 0.077 | 1.30 | 0.21 | 1.60 | 0.77 | 0.163 | 1.26 | 0.10 | 1.40 | 1.13 | 0.082 |
| SW | Stem width (cm) | 0.20 | 0.03 | 0.27 | 0.15 | 0.124 | 0.10 | 0.02 | 0.13 | 0.06 | 0.154 | 0.480* | 0.24 | 0.04 | 0.32 | 0.16 | 0.177 | 0.25 | 0.03 | 0.31 | 0.22 | 0.112 |
| 1InL | 1st Internode length (cm) | 44.20 | 6.29 | 58.20 | 26.70 | 0.142 | 16.00 | 3.44 | 25.50 | 9.00 | 0.215 | 0.253 | 39.40 | 6.75 | 47.40 | 23.80 | 0.171 | 57.30 | 4.52 | 64.10 | 52.00 | 0.079 |
| 2InL | 2nd Internode length (cm) | 25.90 | 3.10 | 33.10 | 14.90 | 0.120 | 10.70 | 2.49 | 19.30 | 4.80 | 0.233 | 0.366 | 21.40 | 3.26 | 26.20 | 13.70 | 0.152 | 23.70 | 1.78 | 26.40 | 20.50 | 0.075 |
| 3InL | 3rd Internode length (cm) | 20.11 | 2.26 | 28.70 | 14.60 | 0.112 | 6.79 | 1.15 | 10.20 | 3.10 | 0.169 | 0.066 | 17.82 | 2.93 | 23.40 | 10.90 | 0.164 | 17.45 | 2.13 | 20.20 | 14.70 | 0.122 |
| 4InL | 4th Internode length (cm) | 18.92 | 2.17 | 23.30 | 12.60 | 0.115 | 5.96 | 1.33 | 13.20 | 0.00 | 0.223 | –0.068 | 16.86 | 3.53 | 21.60 | 6.90 | 0.209 | 15.00 | 2.94 | 18.20 | 11.70 | 0.196 |
| 5InL | 5th Internode length (cm) | 22.14 | 6.36 | 42.80 | 8.13 | 0.287 | 7.21 | 3.70 | 18.20 | 0.00 | 0.513 | 0.302 | 16.26 | 7.75 | 27.17 | 2.10 | 0.477 | 20.13 | 3.95 | 24.40 | 12.20 | 0.196 |
| StL | Stem length (cm) | 131.20 | 14.19 | 165.70 | 89.10 | 0.108 | 46.70 | 6.99 | 62.70 | 29.70 | 0.150 | 0.192 | 115.50 | 25.99 | 182.30 | 57.40 | 0.225 | 133.50 | 5.63 | 143.40 | 127.00 | 0.042 |
| PH | Plant height (cm) | 139.30 | 14.13 | 172.90 | 94.70 | 0.101 | 50.40 | 7.11 | 67.10 | 33.00 | 0.141 | 0.156 | 129.70 | 26.80 | 198.30 | 66.80 | 0.207 | 142.40 | 5.80 | 152.20 | 135.30 | 0.041 |
| SL | Spike length (cm) | 8.07 | 1.11 | 12.50 | 5.60 | 0.138 | 3.67 | 0.68 | 5.80 | 2.40 | 0.185 | 0.267 | 14.14 | 1.73 | 16.90 | 9.40 | 0.122 | 8.90 | 0.82 | 10.10 | 7.30 | 0.092 |
| SN | Number of spike | 10.76 | 2.42 | 17.00 | 3.00 | 0.225 | 80.27 | 21.29 | 128.00 | 48.00 | 0.27 | 0.332 | 6.94 | 1.71 | 10.00 | 3.00 | 0.247 | 3.50 | 0.50 | 4.00 | 3.00 | 0.143 |
| SpN | Number of spilelets | 12.09 | 1.07 | 15.00 | 9.00 | 0.089 | 4.78 | 0.65 | 6.00 | 3.00 | 0.136 | 0.107 | 19.40 | 2.38 | 21.30 | 11.30 | 0.123 | 22.08 | 1.48 | 24.00 | 20.00 | 0.067 |
| SpD | Spikelet density | 1.55 | 0.15 | 1.97 | 1.18 | 0.100 | 1.34 | 0.26 | 2.08 | 0.70 | 0.197 | 0.292 | 1.44 | 0.08 | 1.54 | 1.32 | 0.053 | 2.55 | 0.11 | 2.73 | 2.38 | 0.044 |
| BSpL | Bottom spikelet length (cm) | 1.26 | 0.13 | 1.73 | 0.96 | 0.103 | 1.23 | 0.11 | 1.60 | 0.91 | 0.091 | 0.370 | 1.14 | 0.08 | 1.30 | 1.05 | 0.071 | 1.06 | 0.04 | 1.11 | 1.00 | 0.040 |
| BSpW | Bottom spikelet width (cm) | 0.50 | 0.04 | 0.58 | 0.35 | 0.087 | 0.49 | 0.06 | 0.64 | 0.36 | 0.112 | 0.229 | 0.50 | 0.08 | 0.65 | 0.35 | 0.165 | 0.54 | 0.03 | 0.59 | 0.50 | 0.054 |
| MSpL | Middle spikelet length (cm) | 1.34 | 0.13 | 1.77 | 1.07 | 0.094 | 1.20 | 0.13 | 1.55 | 0.89 | 0.108 | 0.486* | 1.28 | 0.12 | 1.53 | 1.15 | 0.090 | 1.14 | 0.05 | 1.21 | 1.04 | 0.048 |
| MSpW | Bottom spikelet width (cm) | 0.54 | 0.05 | 0.63 | 0.36 | 0.086 | 0.50 | 0.06 | 0.65 | 0.35 | 0.126 | 0.471* | 0.62 | 0.08 | 0.79 | 0.46 | 0.137 | 0.65 | 0.03 | 0.70 | 0.61 | 0.049 |
| TSpL | Top spikelet length (cm) | 1.25 | 0.12 | 1.63 | 1.04 | 0.096 | 0.93 | 0.21 | 1.46 | 0.35 | 0.224 | 0.567** | 1.06 | 0.11 | 1.33 | 0.82 | 0.107 | 1.01 | 0.09 | 1.14 | 0.87 | 0.092 |
| TSpW | Top spikelet width (cm) | 0.40 | 0.05 | 0.50 | 0.25 | 0.133 | 0.38 | 0.10 | 0.62 | 0.16 | 0.269 | 0.375 | 0.39 | 0.06 | 0.50 | 0.28 | 0.146 | 0.52 | 0.05 | 0.61 | 0.45 | 0.096 |
| BLWR | Length-width-ratio of bottom spikelet | 2.55 | 0.30 | 3.86 | 1.96 | 0.116 | 2.51 | 0.25 | 3.34 | 1.85 | 0.099 | 0.059 | 2.36 | 0.52 | 3.77 | 1.66 | 0.219 | 1.96 | 0.14 | 2.13 | 1.74 | 0.073 |
| MLWR | Length-width-ratio of middle spikelet | 2.52 | 0.31 | 3.83 | 2.08 | 0.121 | 2.40 | 0.26 | 3.18 | 1.83 | 0.107 | 0.185 | 2.12 | 0.41 | 2.99 | 1.48 | 0.191 | 1.77 | 0.11 | 1.90 | 1.61 | 0.063 |
| TLWR | Length-width-ratio of top spikelet | 3.17 | 0.49 | 5.05 | 2.44 | 0.153 | 2.52 | 0.42 | 3.95 | 1.67 | 0.167 | 0.297 | 2.79 | 0.46 | 3.70 | 1.98 | 0.165 | 1.97 | 0.14 | 2.15 | 1.78 | 0.070 |
| BAL | Bottom awn length | 4.53 | 1.09 | 7.97 | 2.67 | 0.241 | 2.57 | 0.65 | 5.10 | 1.20 | 0.251 | 0.674*** | 4.18 | 1.07 | 6.10 | 2.63 | 0.256 | 9.64 | 2.19 | 12.20 | 7.10 | 0.227 |
| MAL | Middle awn length | 6.85 | 1.49 | 10.40 | 3.57 | 0.218 | 3.39 | 0.62 | 5.10 | 2.10 | 0.183 | 0.643*** | 7.64 | 1.22 | 9.40 | 4.97 | 0.159 | 13.49 | 0.36 | 14.10 | 13.07 | 0.026 |
| TAL | Top awn length | 7.90 | 1.00 | 10.40 | 5.43 | 0.126 | 3.12 | 0.67 | 6.00 | 2.00 | 0.216 | 0.482* | 7.15 | 1.09 | 9.00 | 4.87 | 0.153 | 11.51 | 0.79 | 12.70 | 10.13 | 0.068 |
| AS | Grain area size (mm2) | 18.34 | 3.90 | 34.00 | 7.79 | 0.213 | 9.25 | 3.06 | 18.70 | 2.35 | 0.331 | 0.518** | 16.49 | 2.73 | 23.80 | 9.20 | 0.166 | 15.78 | 1.94 | 19.40 | 11.42 | 0.123 |
| PL | Perimeter length of grain (mm) | 21.3 | 2.11 | 29.10 | 15.20 | 0.099 | 14.10 | 2.34 | 21.20 | 6.70 | 0.166 | 0.593** | 19.60 | 1.55 | 23.90 | 14.90 | 0.079 | 18.90 | 1.02 | 21.00 | 16.30 | 0.054 |
| GL | Grain length (mm) | 9.01 | 0.91 | 12.56 | 6.27 | 0.101 | 5.79 | 0.98 | 8.84 | 2.70 | 0.169 | 0.649*** | 8.13 | 0.70 | 9.89 | 6.06 | 0.086 | 8.03 | 0.42 | 8.73 | 6.86 | 0.052 |
| GW | Grain width (mm) | 2.64 | 0.40 | 4.00 | 1.49 | 0.150 | 2.08 | 0.43 | 3.46 | 1.06 | 0.204 | 0.325 | 2.72 | 0.34 | 3.79 | 1.83 | 0.126 | 2.68 | 0.27 | 3.35 | 2.08 | 0.100 |
| GLWR | Length-width-ratio of grain | 3.47 | 0.47 | 6.06 | 2.18 | 0.134 | 2.84 | 0.45 | 4.64 | 1.89 | 0.160 | 0.333 | 3.03 | 0.42 | 4.39 | 2.20 | 0.137 | 3.02 | 0.26 | 3.91 | 2.55 | 0.087 |
| CS | Circurarity | 0.506 | 0.05 | 0.68 | 0.30 | 0.100 | 0.58 | 0.06 | 0.72 | 0.39 | 0.100 | 0.415** | 0.54 | 0.05 | 0.65 | 0.37 | 0.102 | 0.55 | 0.03 | 0.62 | 0.46 | 0.063 |
