# Supplementary material for: Phenotypic effects of the U-genome variation in nascent synthetic hexaploids derived from interspecific crosses between durum wheat and its diploid relative Aegilops umbellulata
Source: PLoS One. 2020 Apr 2;15(4):e0231129. doi: 10.1371/journal.pone.0231129 (PMC7117738; doi:10.1371/journal.pone.0231129)
Supplement: S3 Table — (DOC) [file pone.0231129.s003.doc]

**S3 Table.** Eigenvectors for PC1, PC2, and PC3 among Ldn and the ABU and ABD hexaploids based on all morphological and spikelet- and grain-related traits examined.


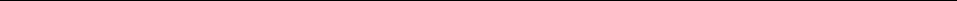


| Traits | PC1 | PC2 | PC3 |
| --- | --- | --- | --- |
|  |  |  |  |
| HD | 0.009 | 0.003 | –0.341 |
| FD | 0.012 | 0.049 | –0.296 |
| FLL | 0.215 | 0.127 | –0.010 |
| FLW | 0.233 | 0.030 | 0.006 |
| LL2 | 0.235 | 0.096 | 0.021 |
| LW2 | 0.234 | 0.042 | –0.006 |
| SW | 0.232 | 0.019 | –0.114 |
| 1InL | 0.016 | –0.291 | 0.078 |
| 2InL | –0.160 | –0.256 | 0.133 |
| 3InL | –0.144 | –0.091 | –0.090 |
| 4InL | –0.139 | –0.245 | 0.036 |
| 5InL | –0.087 | –0.259 | –0.011 |
| StL | –0.104 | –0.329 | 0.048 |
| PH | –0.076 | –0.319 | 0.029 |
| SL | 0.199 | 0.204 | 0.103 |
| SN | –0.233 | –0.106 | 0.080 |
| SpN | 0.271 | 0.074 | –0.001 |
| SpD | 0.112 | –0.220 | –0.173 |
| BSpL | –0.196 | 0.197 | –0.057 |
| BSpW | 0.107 | –0.120 | –0.008 |
| MSpL | –0.167 | 0.210 | 0.016 |
| MSpW | 0.246 | –0.072 | 0.085 |
| TSpL | –0.127 | –0.011 | –0.099 |
| TSpW | 0.105 | –0.201 | –0.007 |
| BLWR | –0.181 | 0.230 | –0.033 |
| MLWR | –0.236 | 0.185 | –0.052 |
| TLWR | –0.190 | 0.205 | –0.078 |
| BAL | 0.020 | 0.055 | –0.305 |
| MAL | 0.135 | 0.111 | –0.256 |
| TAL | 0.053 | –0.042 | –0.293 |
| AS | –0.110 | 0.147 | 0.284 |
| PL | –0.187 | 0.153 | 0.134 |
| GL | –0.194 | 0.127 | 0.097 |
| GW | 0.049 | 0.127 | 0.380 |
| GLWR | –0.197 | –0.011 | –0.280 |
| CS | 0.168 | –0.009 | 0.307 |
|  |  |  |  |
