# Supplementary material for: Phenotypic effects of the U-genome variation in nascent synthetic hexaploids derived from interspecific crosses between durum wheat and its diploid relative Aegilops umbellulata
Source: PLoS One. 2020 Apr 2;15(4):e0231129. doi: 10.1371/journal.pone.0231129 (PMC7117738; doi:10.1371/journal.pone.0231129)
Supplement: S4 Table — The SKCS data of the other ABU hexaploids were referred to our previous study [31]. (DOC) [file pone.0231129.s004.doc]

**S4 Table.** Grain characters in synthetic hexaploids with the AABBUU genome and Ldn as measured by SKCS.


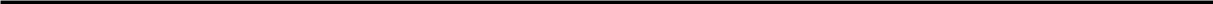


| Sample | hardness |  | weight (mg) |  | diameter (mm) |  | moisture (%) |  |
| --- | --- | --- | --- | --- | --- | --- | --- | --- |
| Ldn | 87.66 | 14.07 | 37.03 | 6.93 | 2.36 | 0.41 | 12.25 | 0.49 |
| Ldn/KU-2932 | 76.21 | 17.19 | 43.79 | 9.43 | 2.50 | 0.37 | 9.98 | 0.37 |
| Ldn/KU-4001 | 76.86 | 16.65 | 42.64 | 11.89 | 2.46 | 0.42 | 10.05 | 0.36 |
| Ldn/KU-4006 | 63.41 | 14.78 | 30.48 | 9.18 | 1.91 | 0.33 | 10.11 | 0.31 |
| Ldn/KU-4007 | 67.56 | 14.29 | 46.75 | 14.15 | 2.47 | 0.51 | 9.45 | 0.45 |
| Ldn/KU-4010 | 60.27 | 15.56 | 35.53 | 10.88 | 2.14 | 0.45 | 9.66 | 0.31 |
| Ldn/KU-4017 | 71.87 | 15.55 | 51.89 | 11.00 | 2.83 | 0.38 | 9.68 | 0.42 |
| Ldn/KU-4024 | 71.55 | 15.05 | 44.01 | 9.87 | 2.50 | 0.36 | 9.56 | 0.46 |
| Ldn/KU-4035 | 75.09 | 15.43 | 37.14 | 8.30 | 2.28 | 0.36 | 9.64 | 0.26 |
| Ldn/KU-4039 | 88.01 | 16.20 | 45.78 | 11.93 | 2.51 | 0.40 | 10.84 | 0.32 |
| Ldn/KU-4043 | 77.01 | 16.57 | 36.79 | 10.41 | 2.27 | 0.40 | 9.57 | 0.40 |
| Ldn/KU-4046 | 62.66 | 15.70 | 33.83 | 12.51 | 2.08 | 0.50 | 9.99 | 0.43 |
| Ldn/KU-4068 | 59.77 | 14.45 | 36.37 | 9.47 | 2.17 | 0.37 | 9.78 | 0.55 |
| Ldn/KU-4070 | 57.40 | 13.58 | 42.41 | 12.66 | 2.34 | 0.49 | 10.25 | 0.56 |
| Ldn/KU-4074 | 68.38 | 17.51 | 35.53 | 9.66 | 2.14 | 0.38 | 9.94 | 0.56 |
| Ldn/KU-4075 | 74.18 | 14.96 | 33.21 | 9.05 | 2.07 | 0.42 | 10.46 | 0.39 |
| Ldn/KU-4081 | 72.57 | 11.28 | 37.70 | 9.37 | 2.25 | 0.31 | 10.27 | 0.40 |
| Ldn/KU-4109 | 68.22 | 17.45 | 34.88 | 8.57 | 2.09 | 0.37 | 10.06 | 0.30 |
| Ldn/KU-12198 | 75.18 | 16.40 | 38.94 | 10.83 | 2.26 | 0.36 | 9.94 | 0.25 |
| Ldn/KU-12200 | 59.15 | 21.49 | 30.04 | 8.59 | 1.91 | 0.45 | 10.31 | 0.58 |

The SKCS data of the other ABU hexaploids were referred to our previous study [31].
