# Supplementary figures and images for: Phenotypic effects of the U-genome variation in nascent synthetic hexaploids derived from interspecific crosses between durum wheat and its diploid relative Aegilops umbellulata
Source: PLoS One. 2020 Apr 2;15(4):e0231129. doi: 10.1371/journal.pone.0231129 (PMC7117738; doi:10.1371/journal.pone.0231129)

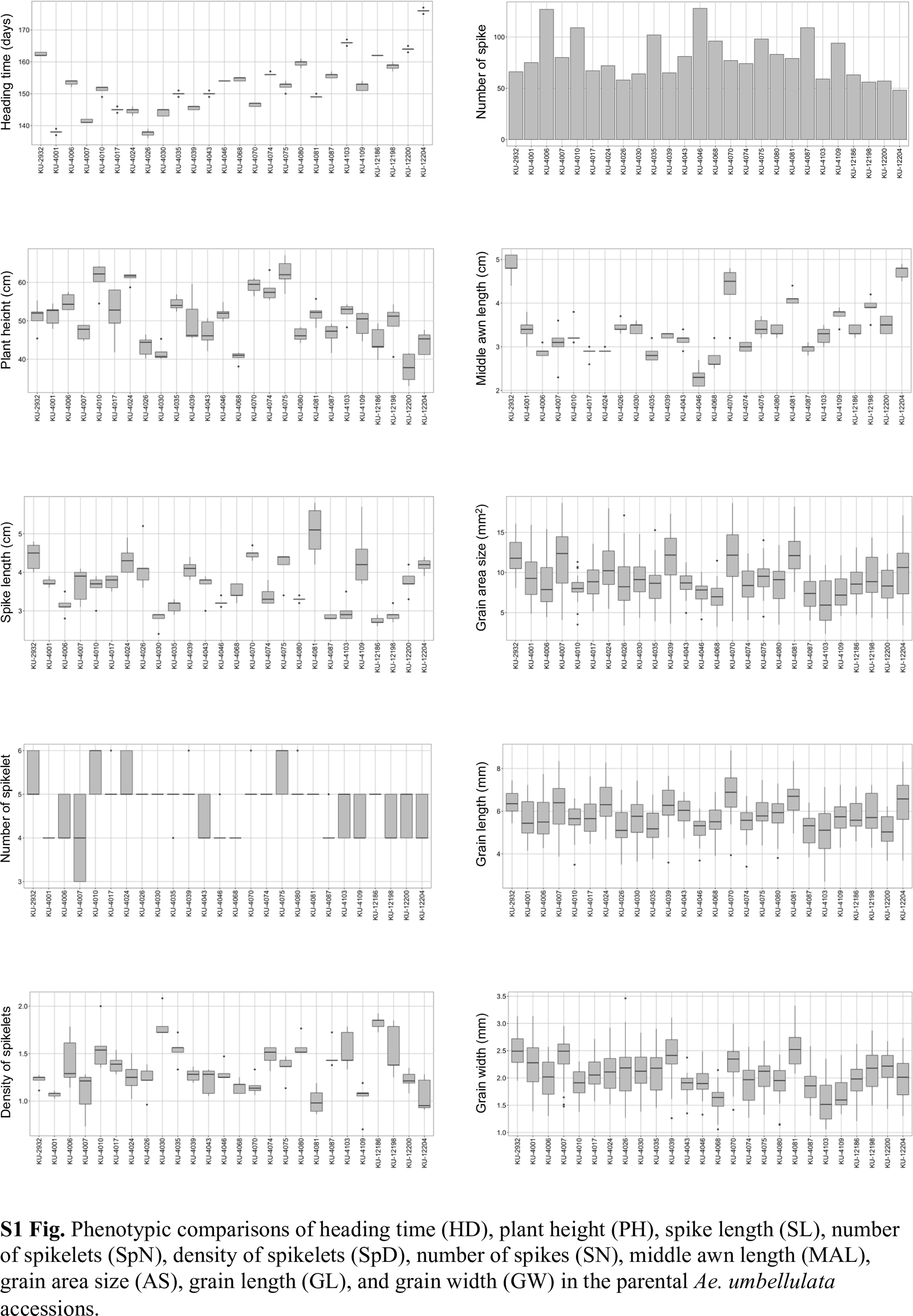

Supplement: S1 Fig — (TIF) [file pone.0231129.s005.tif]

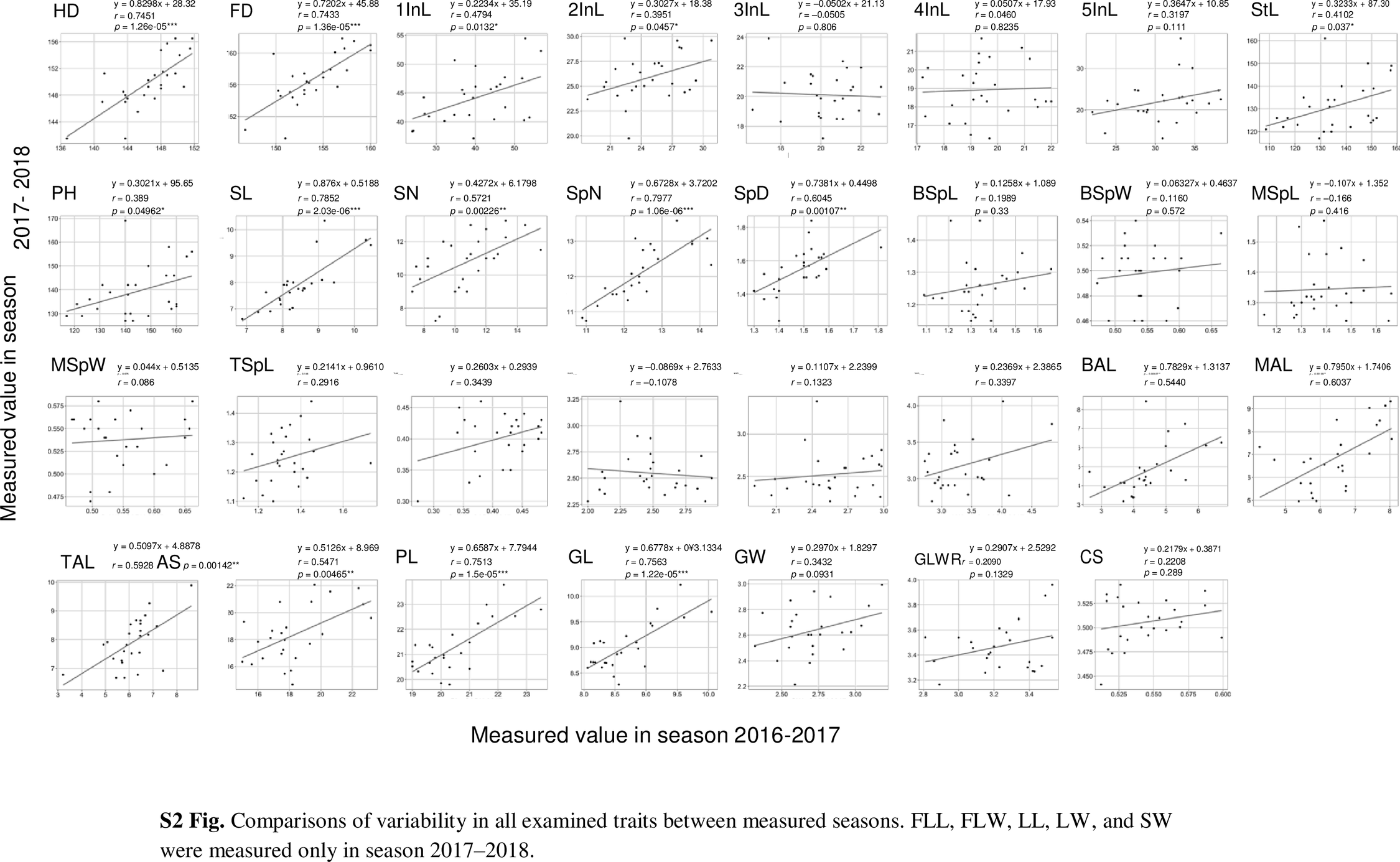

Supplement: S2 Fig — FLL, FLW, LL, LW, and SW were measured only in season 2017–2018. (TIF) [file pone.0231129.s006.tif]

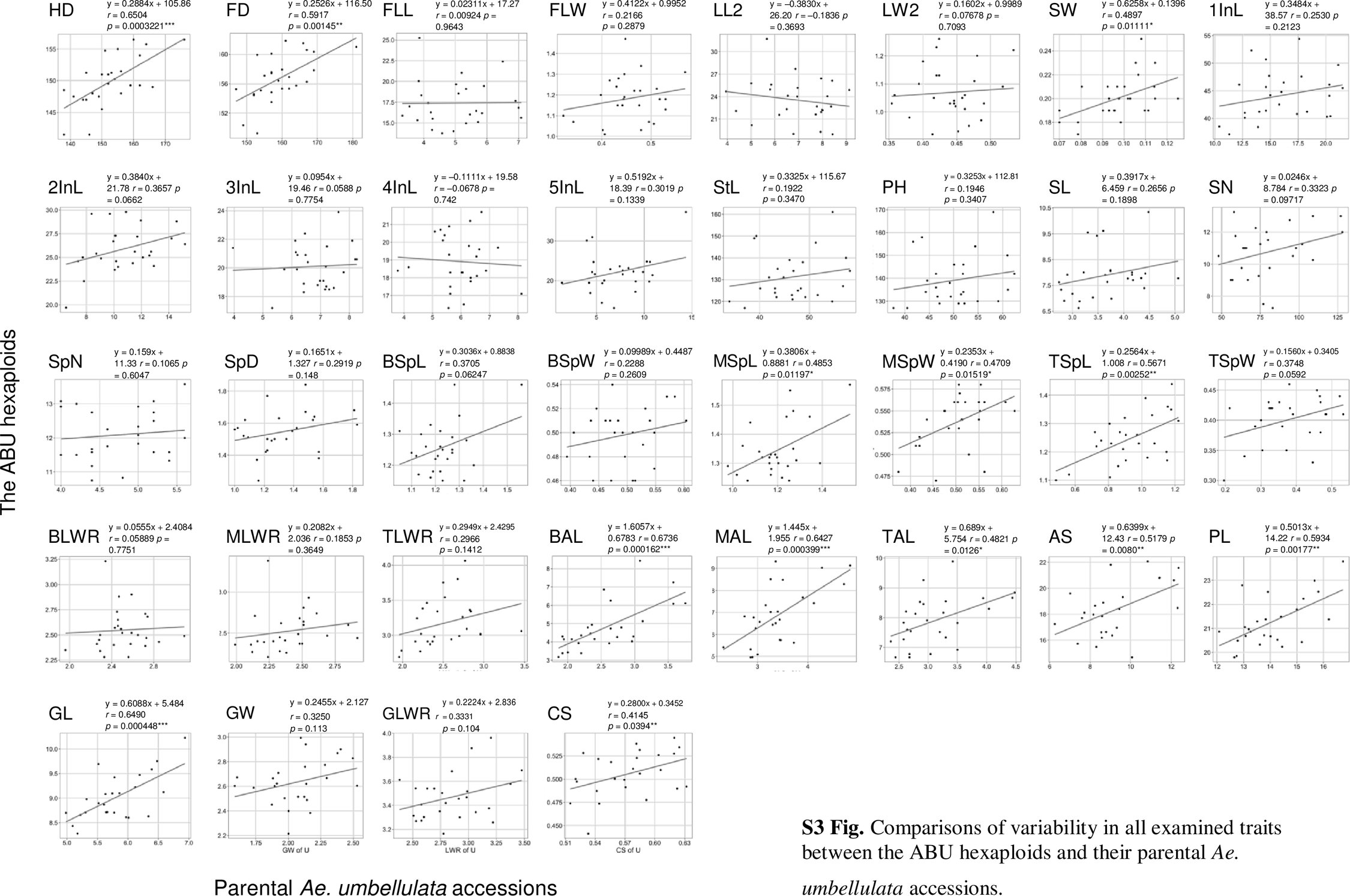

Supplement: S3 Fig — (TIF) [file pone.0231129.s007.tif]

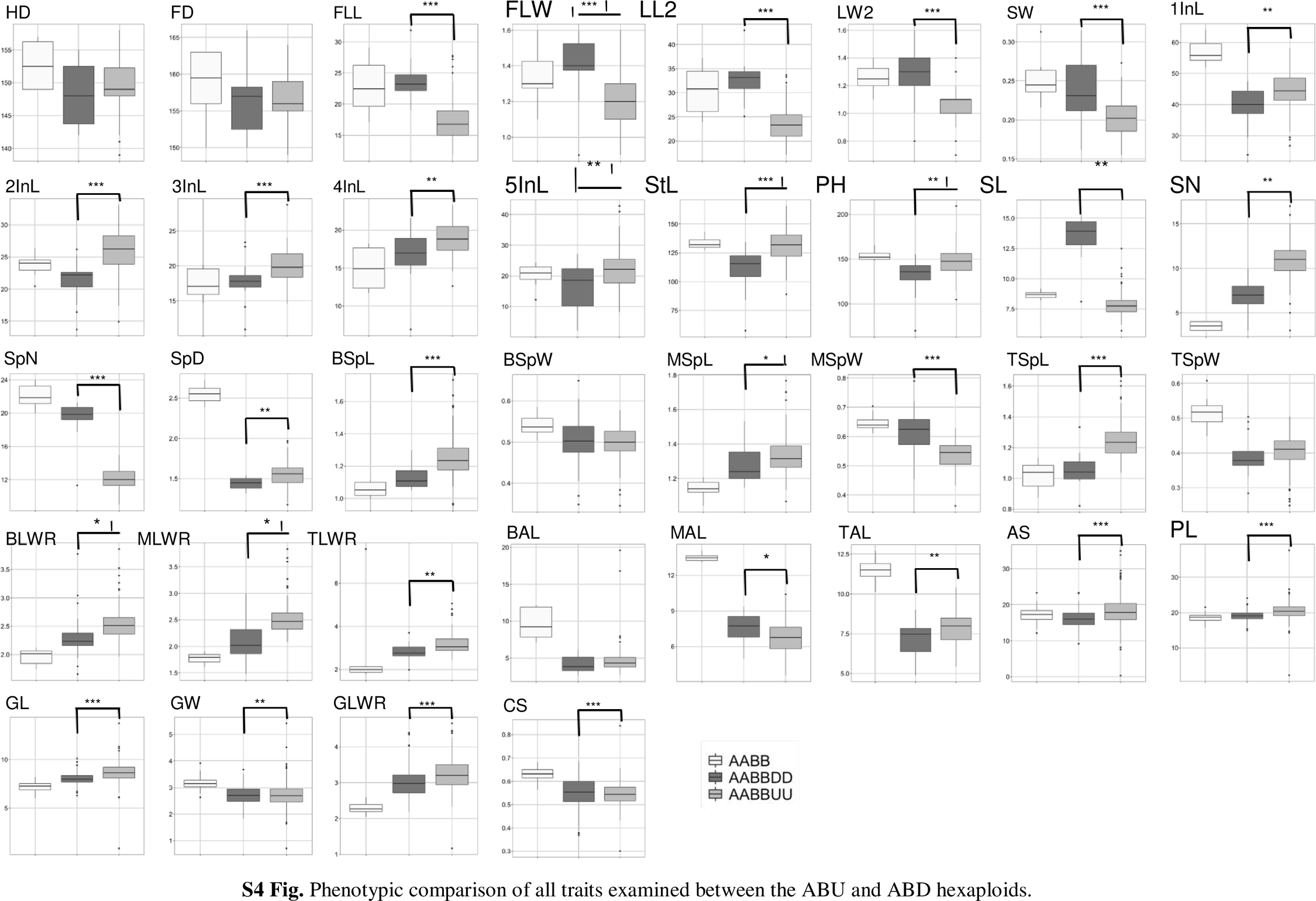

Supplement: S4 Fig — (TIF) [file pone.0231129.s008.tif]
