# Supplementary material for: Phenotypic effects of the U-genome variation in nascent synthetic hexaploids derived from interspecific crosses between durum wheat and its diploid relative Aegilops umbellulata
Source: PLoS One. 2020 Apr 2;15(4):e0231129. doi: 10.1371/journal.pone.0231129 (PMC7117738; doi:10.1371/journal.pone.0231129)
Supplement: Attachment — Submitted filename: ReponseToReviewer-Okada et al-PLoSO.docx [file pone.0231129.s009.docx]

PONE-D-20-03425

**Phenotypic effects of the U-genome variation in nascent synthetic hexaploids derived from interspecific crosses between durum wheat and its diploid relative *Aegilops umbellulata***

Dear Editors and reviewers,

Thank you very much for the reviewers’ kind comments and suggestions to our manuscript.

We examined all of the comments, and revised our manuscript according to the comments as followed.

Improved sentences and words were marked by red color in text and tables.

In addition, we fit the format of our manuscript to the PLoS ONE’s style requirements. Our gel blot images are reported in a submission’s figures but not in Supporting Information files.

To Reviewer #1,

Thank you very much for your understanding our article and for giving us polite comments.

1. The resolution on several of the figures is low, this needs to be improved for them to be of value. In particular, Figures 1, 5, 6, and 8 need work. Additionally, the symbols in the principal component plots (Figure 9) are difficult to see and need to be enlarged.

**<response>** We confirmed the quality of submitted figures (original tif files). Their quality was high. In addition, we improved Fig. 9 with enlarged the plotted symbols.

2. I believe it is PLOS one policy to have abbreviations defined when they first appear in text. Considering the large number of abbreviations in this study, perhaps the supplementary table with all abbreviations could be placed in the materials and methods.

**<response>** We included the abbreviation list for examined traits in S2 Table of Supporting information. In addition, a following sentence was inserted in the second section of M&M; ‘Abbreviations of the examined traits are listed in S2 Table’.

3. p. 5, line 107: It is unclear to me what “representative” means here. Please elaborate on the criteria used to select the synthetic AABBDD hexaploid lines.

**<response>** We omitted ‘representative’, and added one following sentence; ‘These four ABD hexaploids showed various heading/flowering time, and did not exhibit any growth abnormalities [37,39]’ to explain the characteristics of these four lines.

4. p. 7, line 164: The word “repeatedly” can be removed here since it is implied if the same phenotypic traits were measured in both growing seasons.

**<response>** We omitted ‘repeatedly’.

5. p. 8, lines 165-167: This would read better if you discussed the number of plants per line first, then the number of plants per pot.

**<response>** We exchanged the two sentences.

6. p. 8, line 173: The “seed related” traits measured using SmartGrain software should be listed here.

**<response>** We inserted the seed-related traits as followed; ‘The seed-related traits, Grain area size (AS), Perimeter length of grain (PL), Grain length (GL), Grain width (GW), Length-width-ratio of grain (GLWR) and Circularity (CS), were measured using *SmartGrain* software ver. 1.2 [41]’.

7. p. 11, line 243: Can an explanation for no significant correlation for plant height between seasons be provided. In my experience this is an unusual result for wheat.

**<response>** This question is difficult to answer. Little effect of the pollen parents was observed on the plant height under the hexaploid synthetic backgrounds. This means that plant height could be affected by the AB parental cultivar. Therefore, we think that the unusual result is due to the Langdon’s characteristics. Langdon is sensitive to the environmental growth conditions.

8. p. 16, line 362: “data-based” should probably be “data-base”.

**<response>** We changed this tem to ‘the RNA-seq-based’.

9. p. 17, line 385: “mask” would be a clearer word than “cover”.

**<response>** We changed from ‘cover’ to ‘mask’.

10. p.17, lines 386-387: The sentence “Sometimes strong major genes hide the effects of minor quantitative trait loci in regard to morphological phenotypes.” should be supported with citations or removed.

**<response>** We added a reference; Nguyen et al. (2015).

We believe that the revised manuscript is now suitable for publication. We look forward to hearing from you at your earliest convenience.

Yours sincerely,

Shigeo Takumi

(Corresponding author)
